# The Preparation and Characterization of Chitooligosaccharide–Polylactide Polymers, and In Vitro Release of Microspheres Loaded with Vancomycin

**DOI:** 10.3390/jfb13030113

**Published:** 2022-08-04

**Authors:** Jiaxin Li, Ruimin Tang, Penghao Zhang, Minglong Yuan, Hongli Li, Mingwei Yuan

**Affiliations:** National and Local Joint Engineering Research Center for Green Preparation Technology of Biobased Materials, Yunnan Minzu University, Kunming 650500, China

**Keywords:** chitooligosaccharide–polylactide polymers, vancomycin, drug microspheres, biomedical materials, sustained release

## Abstract

Drug-loaded microspheres are an ideal bone tissue delivery material. In this study, a biodegradable Schiff base chitosan–polylactide was used as the encapsulation material to prepare drug-loaded microspheres as biocompatible carriers for controlled vancomycin release. In this regard, Schiff base chitosan was prepared by the Schiff base method, and then different proportions of the Schiff base chitosan–polylactide polymer were prepared by ring-opening polymerization. Drug-loaded microspheres were prepared by the W/O emulsion method, and the polymers and polymer microspheres were characterized and studied by NMR, IR, and antibacterial methods. The drug loading and release rates of microspheres were determined to investigate the drug loading, encapsulation efficiency, and release rate of drug microspheres at different ratios. In this study, different proportions of Schiff base chitosan–polylactic acid materials are successfully prepared, and vancomycin-loaded microspheres are successfully prepared using them as carriers. This study proves that the materials have antibacterial activities against Staphylococcus aureus and Escherichia coli. The particle size of drug-loaded microspheres was below 10 μm, and the particle size decreased with decreasing molecular weight. The obtained results show that 1:100 microspheres have the highest drug-loading and encapsulation efficiencies, the drug-loaded microspheres have no burst release within 24 h, and the release quantity reaches more than 20%. After 30 days of release, the release amounts of 1:10, 1:20, 1:40, 1:60, and 1:100 drug-loaded microspheres were 64.80 ± 0.29%, 54.43 ± 0.54%, 44.60 ± 0.43%, 42.53 ± 0.40% and 69.73 ± 0.45%, respectively, and the release amount of 1:100 was the highest.

## 1. Introduction

Vancomycin is a tricyclic glycopeptide and an antibiotic. Methicillin-resistant Staphylococcus aureus (MRSA) is the most common pathogen in hospital infections. Vancomycin has related antibiotic activity against Gram-positive pathogens, including MRSA [1]. With the intersection and composite of polymer materials science and modern medicine, the potential application of polymer materials as drug-controlled release carriers has become one of the hotspots of current research [2]. Scientific research shows that polymer materials can be used as drug-controlled release carriers. They not only improve the continuous and stable delivery of drug molecules, but also maintain efficiency within the concentration range of drugs in the blood, and they are also safe, effective, and convenient biopolymer materials [3,4,5]. Chitooligosaccharide, as a natural cationic polymer, has attracted wide attention from researchers due to its unique antibacterial, nontoxic, biodegradable, and biocompatible properties [6,7,8]. The hydrogen bonds inside and outside the chito of chitooligosaccharides produce a compact structure, with ultrahigh water solubility [9,10], and biological activities, such as easy absorption, and anti-inflammation and antitumor properties. Biocompatible polymers and drug-loaded microspheres promote the development of bone regeneration. The combination of functional substances and chitooligosaccharides can effectively improve the utilization rate of chitooligosaccharides, making them more widely used in the treatment of bone diseases [11]. Polylactic acid is considered to be one of the most promising biomedical materials in the twenty-first century. With the continuous development of polymer chemistry, the application and sustained efficacy of polylactic acid drugs will continue to improve [12,13]. The obtained results show that the increase in chitosan content s conducive to enhancing the cell adhesion, proliferation, and metabolic activity of the composite surface and does not affect the biocompatibility of the biocomposites [14]. Therefore, the graft copolymer performance of chitosan oligosaccharide and polylactic acid can be improved in theory, which is of great significance for subsequent research.

Traumatic osteomyelitis or infectious bone defects mostly increase the difficulty of treatment because of the risk of infection in the treatment of multiple loading. Conventional oral or intravenous antibiotic therapy, due to high doses of drugs, easily causes adverse reactions and poor local treatment. The desired antibiotic sustained release carrier material should have a certain drug-loading and stable-release rate. Local administration can enhance drug efficacy while effectively releasing antibiotics to control infection and can effectively avoid adverse effects due to systemic administration [15,16]. Chitooligosaccharides and polylactic acid are widely used in gene- and drug-carrier vectors due to their good biocompatibility and biodegradability [17,18].

In this study, different proportions of the Schiff base chitosan–polylactic acid polymer (N-COS-PLA) are prepared from chitooligosaccharides and lactide. Amino Schiff base is generated by amino as the modification site, and the antibacterial activity of chitosan oligosaccharide is improved to the maximum extent. The characterization and performance of N-COS-PLA are studied by NMR, IR, and X-ray diffraction to explore its antibacterial activity against Gram-negative and Gram-positive bacteria. The application value of chitooligosaccharides is improved, and the application field of chitooligosaccharides is expanded. At the same time, chitooligosaccharide hydroxyl groups are connected to polylactic acid to prepare polymer materials, and vancomycin is wrapped to prepare drug-loaded microspheres. The effects of different proportions of polymers on drug loading, encapsulation efficiency, and release amount are studied.

## 2. Experimental

### 2.1. Material

Chitooligosaccharides (CAS: 148411-57-8) (deacetylation degree 90%, molecular weight ≤2000 kDa) were purchased from Shanghai McLin Co., Ltd. (Shanghai, China). Lactide was provided by Fengyuan Co., Ltd. (Hefei, China). All reagents in the experiment were analytically pure and were obtained from Tianjin Damao Co., Ltd. (Tianjin, China). All chemical reagents were not purified further. Staphylococcus aureus and Escherichia coli were obtained from Luwei Technology Co., Ltd. (Shanghai, China).

### 2.2. Synthesis

#### 2.2.1. Synthesis of COS-PLA

The procedure was as follows. Take a certain amount of chitooligosaccharides (COS), dissolved in de-ionized water; add the same amount of methanol solution to induce swelling for 1 h; then, add benzaldehyde and methanol 1:10 mixed solution and continue the reaction at 65 °C for 3 h. After the reaction, cool the solution to room temperature. Using a 5% NaOH solution, adjust the pH to alkaline. Perform the centrifugal collection of solid precipitation. Wash the precipitate with anhydrous ethanol three times. Vacuum dry the precipitate at 45 °C for 24 h. The obtained product is the amino Schiff base chitooligosaccharides (N-COS) [19].

#### 2.2.2. Synthesis of N-COS-PLA

**Figure 1:**
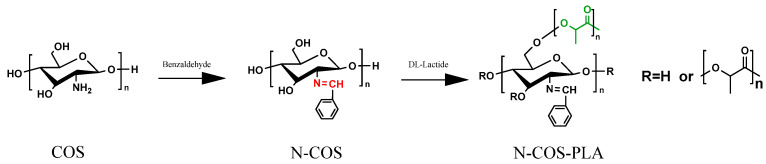
Reaction chemistry for the synthesis of N-COS and N-COS-PLA.

N-COS and lactide were added to a small amount of dry DMSO solution at room temperature according to different molar mass ratios (1:10, 1:20, 1:40, 1:60, 1:100). The catalyst stannous octoate with a mass fraction of 0.1% was added. Before addition, N_2_ was replaced several times; the reaction temperature was 140 °C, and the reaction time was 8 h. After the reaction, the product was cooled to room temperature and then precipitated by adding anhydrous ethanol. The product was obtained by centrifugation. After vacuum drying at 45 °C for 24 h, different proportions of N-COS-PLA were obtained. The reaction synthesis route is shown in Figure 1 [20].

### 2.3. Preparation of N-COS-PLA Drug-Loaded Microspheres

The vancomycin-loaded microspheres were prepared by the emulsion solvent evaporation method. The appropriate amount of vancomycin was fully dissolved in 5 mL of de-ionized water to prepare the vancomycin solution. The vancomycin solution was dripped into a DCM solution containing composite materials at a high speed of 29,000 rpm/min in a 0 °C ice water bath environment (the water–oil concentration ratio was 1:2). After dripping, the colostrum was formed by continuous high-speed stirring for 5 min, and the colostrum was added to 2% PVA aqueous solution. After dripping, the colostrum was stirred and emulsified at a high speed to form a multiple emulsion. The multiple emulsion was rapidly transferred into an aqueous solution containing 15% isopropanol and stirred at room temperature to completely remove the organic solvent. Solid precipitation was collected and freeze-dried at −50 °C for 24 h to obtain powdered microspheres [21].

### 2.4. Scanning Electron Microscope (SEM) Observation

The morphology of vancomycin-loaded drug microspheres with different ratios of N-COS-PLA was observed by scanning electron microscopy (S-3000N, Hitachi, Tokyo, Japan). The microspheres were numbered according to the different preparation conditions, and the micro-sphere solution was dropped on the clean glass slide and dried naturally. The glass slides were pasted on the carrier table by conductive adhesive, sprayed with gold, and the morphology was observed [20].

### 2.5. Nuclear Magnetic Test (NMR)

The ^1^HNMR spectra of the copolymer were determined by NMR (BRUKER 400 MHz, Karlsruhe, Germany). The determination temperature was 25 °C, and the solvent of COS was D_2_O. The solvent of N-COS was DMSO-d_6_; the solvent of N-COS-PLA was CDCL_3_. The molecular weight of the copolymer was determined by gel chromatography (Model GPC-Waters2414, Waters, Milford, MA, USA) to determine the structure and molecular weight distribution of the copolymer. A comprehensive analysis of the characterization results can determine whether the copolymerization was complete and the residual amount of raw materials [22].

### 2.6. Fourier Infrared Test (FT-IR)

COS, N-COS, and N-COS-PLA were recorded by a Fourier transform infrared spectrometer (NicoltAvatar 360 Beijing, China). Fourier transform infrared spectroscopy was performed at room temperature in the frequency range of 4000–4500 cm^−1^. The scanning number was 100 times, and the resolution was 4 cm^−1^. The infrared spectrum of the powder was obtained with a KBr sheet [23].

### 2.7. X-ray Diffraction Test (XRD)

An XRD/max2200 X-ray diffractometer (Rigaku, Tokyo, Japan) was operated at a 40 kV voltage and 40 mA current. COS, N-COS, and N-COS-PLA were analyzed at room temperature by scanning 2θ between 10° and 60° in 45 steps for 10 s [24].

### 2.8. Thermogravimetric Test (TGA)

Thermogravimetric curves of COS, N-COS, and N-COS-PLA were determined by a thermogravimetric analyzer (TG209F1, Netzsch, Bavaria, Germany). Test conditions: heating temperature range: 50–450 °C; N_2_ flow rate: 20 mL/min; heating rate: 10 °C/min [25].

### 2.9. Solubility of Materials

COS, N-COS, and N-COS-PLA were dissolved in 10 organic solvents, including water, ethanol, methanol, DCM, EA, petroleum ether, acetone, DMF, DMSO, and acetic acid. A certain amount of material was added to the abovementioned solvent at room temperature, and the dissolution of substances was observed 24 h after ultrasound treatment.

### 2.10. Antibacterial Property of Materials

The sensitivity of different proportions of polymers to Escherichia coli and Staphylococcus aureus was studied by the filter paper method. First, LB solid medium was configured and cooled to solidification, and 100 μL of bacterial suspension with a certain concentration was evenly coated on the medium surface. The filter paper (10 mm) was soaked in different proportions of polymer solution and sterilized under ultraviolet light. The filter paper was evenly attached to the surface of the culture medium, pre-diffused for 2 h at 4 °C, and cultured in a 37 °C incubator for 24 h. The diameter of the inhibition zone was measured and the average value of each sample was taken three times [26].

### 2.11. Particle-Size Distribution of Drug-Loaded Microspheres

The particle size and distribution of microspheres were determined by a laser particle size analyzer (Mastersizer 2000, Malvern, the United Kingdom). A part of the newly prepared microsphere suspension was diluted to normal light transmission, and the suspension was ultrasonically treated for more than 30 min to make it more evenly dispersed. The suspension was dropped into the sample detection cell of the laser particle-size analyzer, and the particle size and distribution of the microspheres were determined by the particle-size analyzer [27].

### 2.12. Drug Loading and Embedding Ratio

The establishment of the standard curve: vancomycin (25 mg) was accurately weighed and dissolved in a 10 mL volumetric flask to prepare a solution for use as a standard solution. The standard solution was diluted to 25 μg/mL, 50 μg/mL, 100 μg/mL, 200 μg/mL, and 300 μg/mL. The absorbance was determined at each concentration (OD value). The standard curve was established with the the OD value as the ordinate and the concentration (μg/mL) as the abscissa. The linear range was 25–300 μg/mL.

Determination method: A certain amount of microsphere powder was accurately weighed and placed in a centrifuge tube. A small amount of DCM was added to fully dissolve the encapsulation material of the microspheres, and vancomycin embedded in the microspheres was released. Then, a certain volume of PBS was added to dissolve vancomycin, and the encapsulation material N-COS-PLA was precipitated. The solution was centrifuged and a certain volume of supernatant was obtained to determine its OD value. In the experiment, each sample was repeated three times, and the results are expressed as the mean and standard deviation. The measured OD value was taken into the standard curve to calculate the content of vancomycin in the microspheres, and the encapsulation efficiency and drug loading were calculated according to the following formula [28]:

$$\mathrm{Encapsulation}\;\mathrm{rate}\;\left(\%\right)=W_{A}÷W_{B}\times 100\%$$

where *W_A_* and *W_B_* are the drug quality in microspheres and the drug dosage.

$$\mathrm{Loading}\;\mathrm{rate}\;\left(\%\right)=W_{A}÷W_{B}\times 100\%$$

where *W_A_* and *W_B_* are the drug mass in the microspheres and the mass of microspheres.

### 2.13. Drug-Release Profile

The release conditions of microspheres were as follows: 50 mg of microsphere powder was accurately weighed in a 15 mL centrifuge tube, added to 10 mL PBS (pH = 7.4) buffer solution, and continuously released in a 37 °C thermostatic oscillator. At a certain time, the sample was centrifuged, 1 mL of release liquid was extracted from the supernatant, the same amount of fresh release medium PBS buffer solution was added over time, and the constant temperature oscillation was continued. The concentration of vancomycin in a 1 mL release solution was determined by an enzyme-labeled instrument, and the cumulative release of vancomycin in a certain period of time was calculated [29].

## 3. Results and Discussion

### 3.1. SEM of N-COS-PLA Drug-Loaded Microspheres

**Figure 2:**
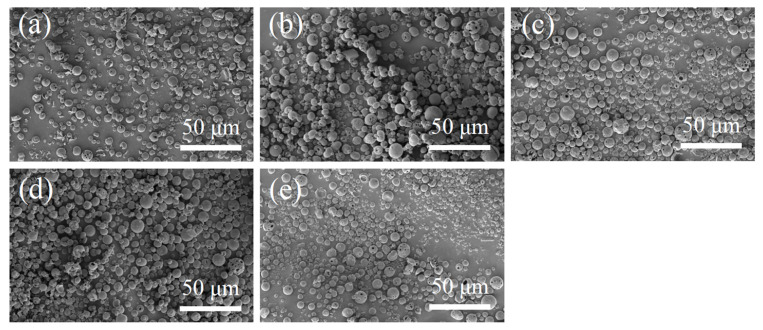
SEM images of N-COS-PLA drug-loaded microspheres. (**a**–**e**) Different proportions of COS:PLA: 1:10, 1:20, 1:40, 1:60, 1:100.

The SEM images of N-COS-PLA composite microspheres are presented in Figure 2. Through the comparative observation of microspheres under the condition of a different water–oil concentration ratio and stirring speed, it was concluded that the microspheres with different ratios had a good forming effect, smooth surface, and were fractured less under the condition of a water–oil concentration ratio of 1:2 and stirring speed of 29,000 rpm/min. Therefore, the microspheres were prepared under this condition to complete the subsequent studies on the particle size, drug loading, encapsulation efficiency, and in vitro sustained-release performance.

### 3.2. NMR of Polylactide-Grafted Schiff Base Chitosan

**Figure 3:**
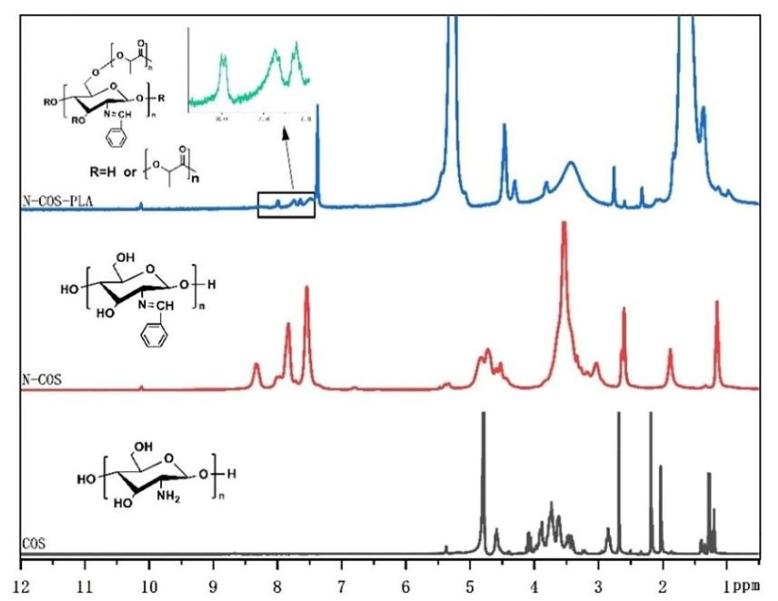
^1^HNMR spectrum of COS, N-COS, and N-COS-PLA.

The ^1^ HNMR results of COS, N-COS, and N-COS-PLA are presented in Figure 3. In the COS ^1^HNMR spectrum, the peak at 1.98 ppm was attributed to -CH_3_ in the N-alkylated glucosamine residue, and singlet H peak for N-acetyl glucosamine was observed at 2.98 ppm; there was a multistate peak at 3.45–4.12 ppm, which was due to the chemical shift of -CH- on the sugar ring and proton hydrogen at 6C. The peak at 4.58 ppm was attributed to H of glucosamine [30]. In addition to the chemical shift of H in COS, the absorption peak of the benzene ring and the chemical shift of -HC=N appeared at 7.73–8.33 ppm and 10.12 ppm, respectively. In the ^1^HNMR analysis of N-COS-PLA, the absorption peaks at 1.67 ppm and 5.27 ppm were the characteristic peaks of CH_3_ and -CH- in the PLA structure, respectively. The ^1^HNMR results show that N-COS and N-COS-PLA are successfully synthesized. With an increase in the molecular weight of the polymer, the characteristic peaks -CH_3_ and -CH- in the structure of PLA in the polymer increased, and the absorption peak intensity of N-COS-PLA at 1.67 ppm and 5.27 ppm increased.

Table 1 presents the GPC results of N-COS-PLA. With an increase in lactide, the Mn value increases, indicating that the molecular weight of polylactic acid also increases. It also shows that the proportion of N-COS in the polymer is declining. When PLA and N-COS were 1:40, the Mn value decreased with the increasing lactide content. Although the molecular weight of the polymer can be changed by changing the content of the catalyst, the content of N-COS is fixed at the same catalyst content, and the molecular weight of polymer N-COS-PLA does not increase with an increase in the amount of lactide.

### 3.3. FT-IR Analysis of the Polymer

**Figure 4:**
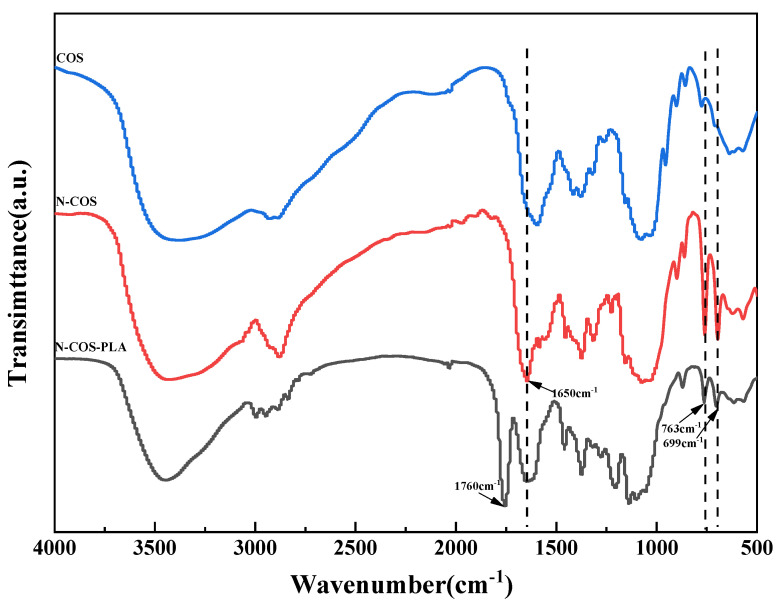
FT-IR spectra of COS, N-COS, and N-COS-PLA.

The infrared characteristic peaks of chitooligosaccharides in the FT-IR spectra (Figure 4) were assigned as follows: there was a characteristic peak at 3422.36 cm^−1^, which can be attributed to the stretching vibrations of -NH and -OH groups and the hydrogen bonds between and outside COS molecules. The weak band of 2923.83 cm^−1^ was attributed to the -CH- stretching of COS. The characteristic peaks at 1628.76, 1519.37, and 1322.79 cm^−1^ were attributed to the carbonyl (C=O) stretching vibration (amide I), amino (N-H) bending vibration (amide II), and amide triple absorption peaks of COS, respectively. The absorption band at 1155.97 cm^−1^ was produced by C-O-C asymmetric stretching [31]. The characteristic peaks of monosubstituted benzene rings were shown in the fingerprint region, and N-COS had strong absorption bands at 763 cm^−1^ and 699 cm^−1^. N-COS-PLA also had strong absorption peaks at 776 cm^−1^ and 705 cm^−1^, presenting a sharp peak. The characteristic peak of C=N corresponding to the Schiff base appeared at 1650 cm^−1^ in FT-IR spectrum, but this peak did not appear in the infrared spectrum of COS. The characteristic peak of the ester group in polylactic acid appeared at 1760 cm^−1^ of N-COS-PLA. The synthesis of N-COS and covalent binding of N-COS-PLA were confirmed.

### 3.4. XRD Analysis

**Figure 5:**
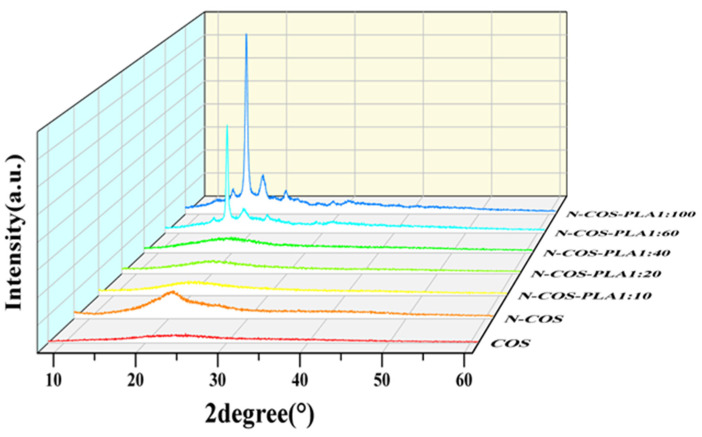
XRD spectra of COS, N-COS, and N-COS-PLA.

Figure 5 shows that there are two diffraction peaks of pure COS at 2θ = 12.51° and 24.93°, which are the characteristic peaks of COS I and II crystallization [32]. By observing the curves of COS and N-COS in the figure, it can be found that a new diffraction peak appears at 2θ = 20.57°, and the peak deformation is sharp. After chitosan oligosaccharide reacted with benzaldehyde to form a Schiff base, the crystallinity changed due to the change in the polymer structure introduced by the benzene ring. At ratios of 1:10, 1:20, and 1:40, the corresponding 2θ values of the maximum diffraction intensity were 19.89°, 20.10° and 19.67°, respectively. These two peaks were not smooth and did not belong to sharp peaks, which confirmed that both polymers contained amorphous states. At ratios of 1:60 and 1:100, the crystallinity of the polymer was enhanced. The α-single crystal diffraction peaks of pure PLA were 16.70° and 19.05°, and its characteristic diffraction peak was 22.32° [33,34]. The characteristic peaks of PLA at 16.67°, 18.90°, and 22.33° were observed in the polymers. Due to the increase in the proportion of polylactic acid, the content of Schiff base chitosan was low, and the peaks at 12.51° and 24.93° became weak or even insignificant. Figure 5 shows that the addition of a benzene ring and the increase in PLA ratio will lead to the transformation of chitooligosaccharides from amorphous to crystalline. This indicates that with increasing crystallinity, the crystal form of the polymer improves.

### 3.5. Thermal Performance

**Figure 6:**
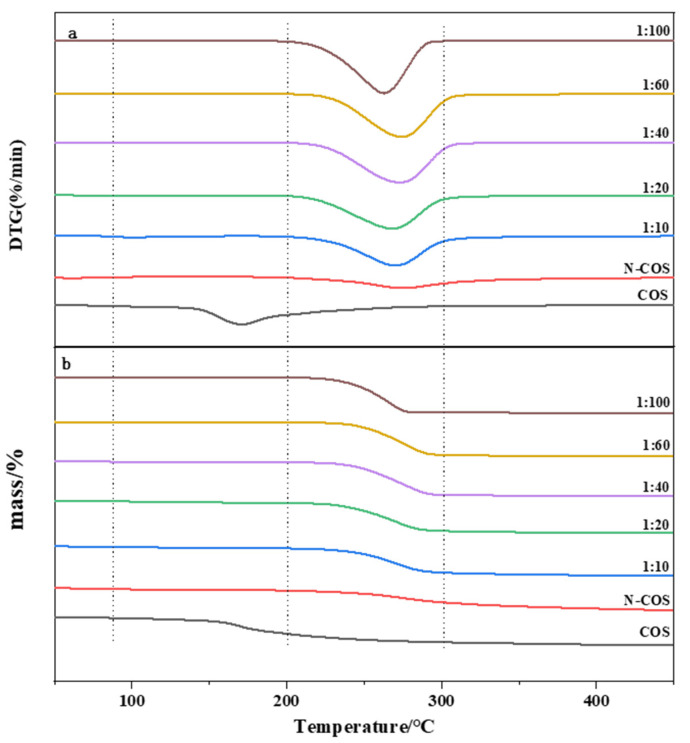
Thermal performance curves of COS, N-COS, and N-COS-PLA. (**a**) DTG curves of COS, N-COS, and N-COS-PLA with different weight ratios (g/g). (**b**) TG curves of COS, N-COS, and N-COS-PLA with different weight ratios (g/g).

The thermal stability of the material was studied by thermogravimetric analysis. Figure 6a presents the DTG curves of COS, N-COS, and N-COS-PLA with different weight ratios. According to the curve analysis, it was observed that both COS and N-COS had two thermogravimetric stages. The first weight-loss stage was mainly due to the evaporation of intramolecular moisture with increasing temperature. The temperature ranges were 55~140 °C and 45~85 °C, and the mass loss rates were 6.94% and 5.6%, respectively. The second weight-loss stage was mainly due to the high-temperature decomposition of the material. The temperature range was 175~350 °C and 195~435 °C; the mass loss rates were 63.58% and 59.93%, and the fastest degradation rate temperature (T_max_) was 202–298 °C. Polymer N-COS-PLA at 1:10 and 1:20 ratios had the first weight-loss stage; the first weight-loss stage was at 75–135 °C and the mass loss rates were 5.13% and 4.94%, perhaps because the molecular weight of the polymer was lower than that at other ratios, and it was slightly soluble in water; thus, the first stage was the evaporation of water molecules. The other ratios only achieved the second weight-loss stage, which was at 195–405 °C. The mass loss rates were 80.25%, 85.05%, 94.43%, 92.90%, and 95.81%, respectively. The second-stage mass loss rates may have been due to the thermal decomposition of the material. Figure 6b presents the TG curves of COS, N-COS, and N-COS-PLA with different weight ratios. The maximum weight-loss temperature of COS was 166.3 °C. The maximum weight-loss temperature of N-COS was 273.9 °C. The maximum weight-loss temperature of the polymer Schiff base chitosan–polylactide was above 260 °C. Both ere higher than the maximum weight-loss temperature of COS and slightly lower than the maximum weight-loss temperature of N-COS. According to the thermal property results, the thermal stability of N-COS-PLA was greater than that of chitooligosaccharide, which may be due to the introduction of chitooligosaccharide by polylactic acid, which enhanced intramolecular hydrogen bonding and led to an increase in thermal stability. Moreover, the thermal stability of the derivatives increased with increasing molecular weight.

### 3.6. The Solubility of Polymers

The dissolution of each substance in organic solvents is presented in Table 2. COS is completely soluble in water, and N-COS is completely insoluble in water compared to COS. The modified chitooligosaccharides were completely dissolved in acetone, DMF, DMSO, and acetic acid. This shows that the introduction of polylactic acid improves the solubility of the polymer in organic solvents.

### 3.7. The Antibacterial Properties of Polymers

**Figure 7:**
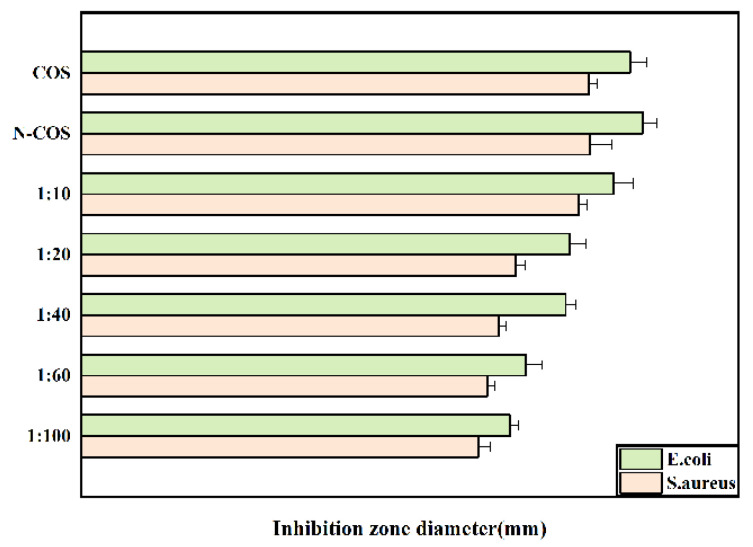
Inhibition-zone diameter of *E.* *coli* and *S.* *aureus* grown with COS, N-COS, and N-COS-PLA (mm).

The inhibition zone diameters of Escherichia coli and Staphylococcus aureus treated with COS, N-COS, and different proportions of N-COS-PLA are presented in Figure 7. As shown in the figure, the inhibition zone decreases with decreasing Schiff base chitosan content after the derivatives are treated with the two bacteria; under the same conditions, the antibacterial circle of derivatives against Escherichia coli is larger than that against Staphylococcus aureus. Therefore, the antibacterial activity of the derivatives against the two bacteria increased with the increase in the C=N group, and the antibacterial activity against Escherichia coli was better than that against Staphylococcus aureus.

### 3.8. Particle-Size Distribution of Drug-Loaded Microspheres

**Figure 8:**
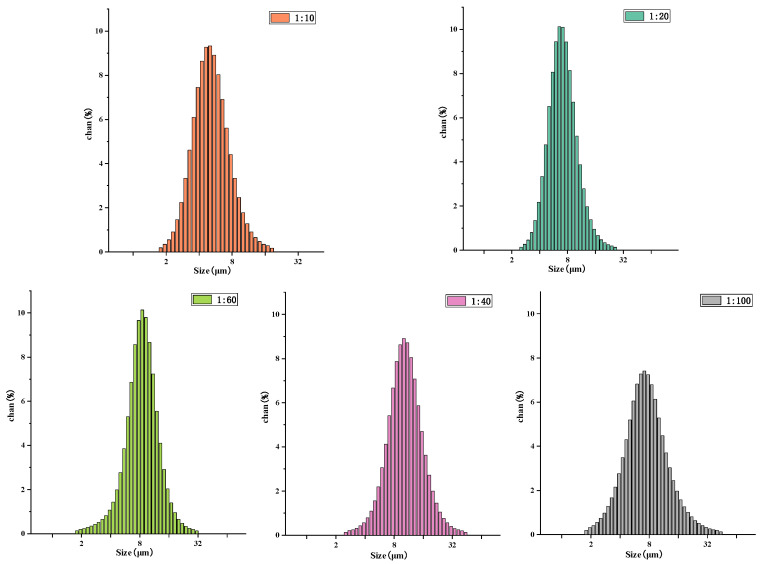
Particle-size distribution of N-COS-PLA drug-loaded microspheres with different proportions.

Figure 8 presents the particle-size distribution of drug-loaded microspheres with different materials. As shown in the figure, the average particle-size distribution of the 1:10 microspheres is 4.30 ± 0.12 µm; the average particle-size distribution of the 1:20 microspheres is 5.36 ± 0.09 µm, the average particle-size distribution of 1:40 microspheres is 9.88 ± 0.11 µm; the average particle-size distribution of the 1:60 microspheres is 8.11 ± 0.06 µm, and the average particle-size distribution of the 1:100 microspheres is 7.01 ± 0.16 µm. It can be observed from the particle-size distributions that the molecular weight of the microsphere-sized polymer increases. This occurs because with a decrease in the PLA ratio, the amount of hydroxyl in the copolymer is greater, which is not conducive to the formation of long chains. The shorter the copolymer molecular chain, the smaller the core–shell diameter and the particle size of the microspheres.

### 3.9. Drug Loading and Embedding Ratio

Encapsulation efficiency and drug loading are two important criteria for evaluating the drug-loading capacity of microspheres. At the same time, encapsulation efficiency and drug loading are affected by many factors in the preparation of microspheres [35], such as the amount of solvent, composition ratio of composite microspheres, drug concentration, and preparation method [36]. The drug-loading ring and encapsulation efficiency of N-COS-PLA microspheres with different ratios are shown in Table 3. In the experimental study, the encapsulation efficiency of the 1:100 microspheres was the highest, while that of the 1:40 and 1:60 microspheres was the lowest. This result indicates that the encapsulation efficiency of microspheres increases with increasing polymer molecular weight within a certain range, but when the polymer molecular weight exceeds a certain range, the encapsulation efficiency of microspheres decreases.

### 3.10. Drug-Release Profile

**Figure 9:**
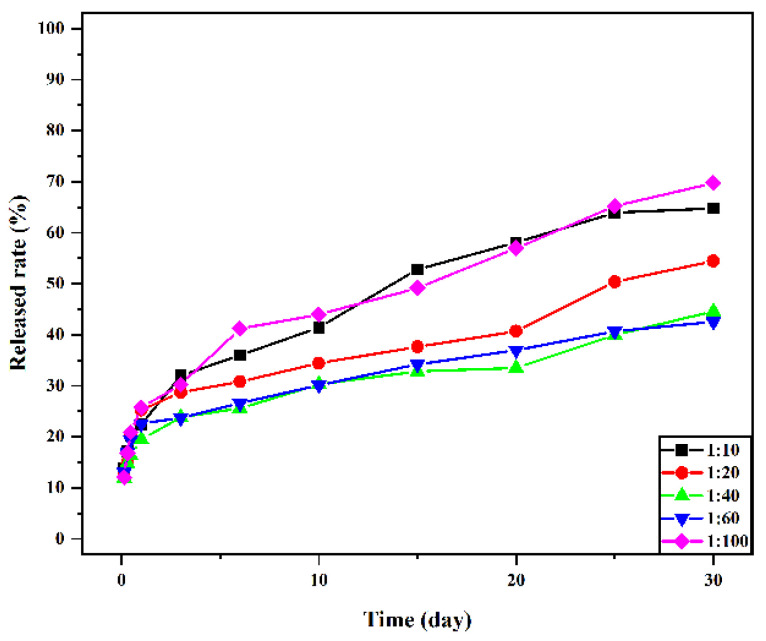
Cumulative release rate of vancomycin in vitro from microspheres with different material ratios.

The drug-release rate was positively correlated with the molecular weight distribution, morphology, drug loading, and encapsulation efficiency of the material [37]. Figure 9 presents the in vitro release curve of drug-loaded microspheres with different material ratios. The figure shows that the release of vancomycin is sustained and stable, and the drug-loaded microspheres have no sudden release within 24 h of drug release. The release amount reached more than 20%, and most of the drugs in the microspheres were dissolved. After 30 days of release, the release amounts of 1:10, 1:20, 1:40, 1:60, and 1:100 drug-loaded microspheres were 64.80 ± 0.29%, 54.43 ± 0.54%, 44.60 ± 0.43%, 42.53 ± 0.40%, and 69.73 ± 0.45%, respectively, and the release amount of 1:100 was the highest. There is no sudden release effect in the release process, which is a very advantageous feature for drug delivery systems. It is conducive to the sustained and stable release of the drug-loaded microspheres in vivo within a certain time range and to prolong the retention time of vancomycin in the human body and the retention time of the effective plasma concentration.

## 4. Conclusions

In the present study, N-COS-PLA polymer materials with different proportions were successfully prepared from chitooligosaccharide and lactide. The introduction of an amino Schiff base can significantly improve the properties of the material, including crystallinity, thermal properties, and solubility. The material had antibacterial effects on Staphylococcus aureus and Escherichia coli, and the molecular weight of PLA had a significant impact on the properties of microspheres. In this study, sustained-release microspheres of vancomycin were prepared by the emulsion crosslinking method. Sustained-release microspheres of vancomycin were prepared by the emulsion crosslinking method. Studies have shown that the particle sizes of drug-loaded microspheres are all below 10 μm, and the particle sizes of microspheres decrease with the decrease in molecular weight. In the experimental study, the drug loading and encapsulation efficiency of 1:100 microspheres were the highest, and there was no burst release within 24 h of drug release. After 30 days of release, the release amounts of 1:10, 1:20, 1:40, 1:60, and 1:100 microspheres were 64.80 ± 0.29%, 54.43 ± 0.54%, 44.60 ± 0.43%, 42.53 ± 0.40%, and 69.73 ± 0.45%, respectively. When the PLA content was the highest, the release amount of 1:100 was the highest. Sustained-release microspheres of vancomycin began to release rapidly in the release medium for 24 h, and the release amount was greater than 20%. The mechanism is that vancomycin hydrochloride molecules contain hydrophilic hydroxyl groups and are water-soluble, which can be combined with water molecules through oxygen bonds and rapidly spread in the solution. In summary, vancomycin sustained-release microspheres showed a good sustained-release effect and antibacterial activity in vitro, which provided a basis for its application in the treatment of bone infection. In the study of vancomycin-loaded materials, the amphiphilic material prepared in this study made the release of vancomycin easier to control and more stable, avoided the side effects of systemic medication, and improved the medication rate.

## Figures and Tables

**Table 1:** Molecular weight and dispersion of N-COS-PLA with different proportions.

Sample	Mn (g/mol)	Mw (g/mol)	Dispersity
1:10	14,945	20,341	1.361
1:20	24,673	32,403	1.311
1:40	45,058	79,738	1.769
1:60	40,818	71,142	1.742
1:100	30,086	42,960	1.427

**Table 2:** Solubility of COS, N-COS, and N-COS-PLA in organic solvents.

	H_2_O	EtOH	MeOH	DCM	EA	PE	PA	DMF	DMSO	HAc
COS	+	−	+	−	−	−	−	±	+	−
COS-N	−	−	−	−	−	−	−	±	+	±
1:10	±	−	+	+	+	−	+	+	+	+
1:20	±	−	±	+	+	−	+	+	+	+
1:40	−	−	±	+	±	−	+	+	+	+
1:60	−	−	±	+	±	−	+	+	+	+
1:100	−	−	−	+	±	−	+	+	+	+

**Table 3:** Drug-loading collar and entrapment efficiency of N-COS-PLA drug-loaded microspheres with different proportions.

Sample	Proportion	Drug Loading (%)	Embedding Rate (%)
1	1:10	8.71 ± 0.01	17.42 ± 0.02
2	1:20	8.36 ± 0.04	16.72 ± 0.08
3	1:40	6.63 ± 0.03	13.26 ± 0.06
4	1:60	6.66 ± 0.03	13.33 ± 0.07
6	1:100	11.28 ± 0.06	22.52 ± 0.012

## Data Availability

The data presented in this study are available on request from the corresponding author.
